# Supplementary material for: Barley Cultivar Sarab 1 Has a Characteristic Region on the Thylakoid Membrane That Protects Photosystem I under Iron-Deficient Conditions
Source: Plants (Basel). 2023 May 26;12(11):2111. doi: 10.3390/plants12112111 (PMC10255597; doi:10.3390/plants12112111)
Supplement: Supplementary file 1 [file plants-12-02111-s001.zip › plants-2344835-supplementary.pdf]

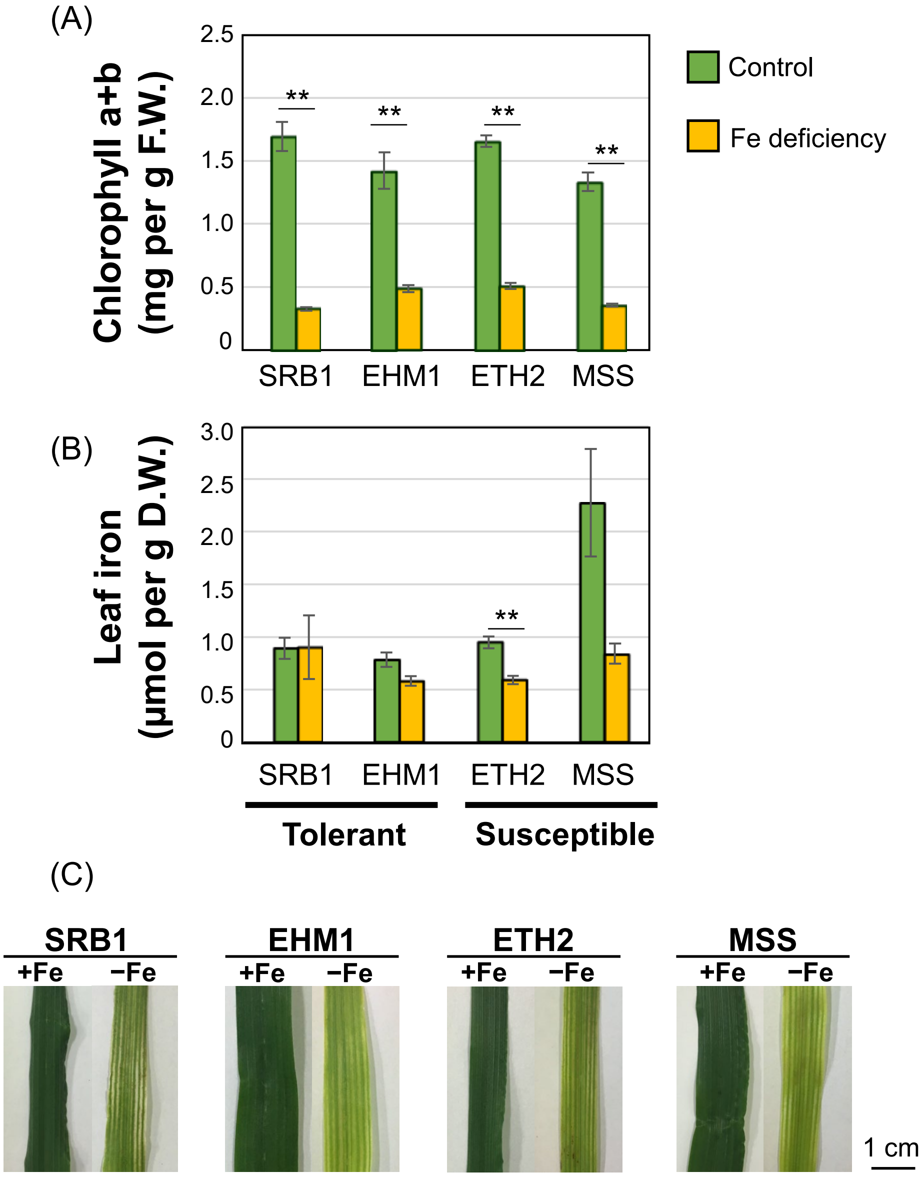

Fig. S1. Comparison of chlorophylls and Fe content of leaves of the varieties used under Fe-sufficient and –deficient conditions. The chlorophyll (A), Fe content (B), and Photograph of a typical newly expanded leaf used in the experiment (C). Data are presented as means and standard errors of triplicate data obtained from independent leaves. Significant differences are shown by Student's t-test (** $p<0.01$).

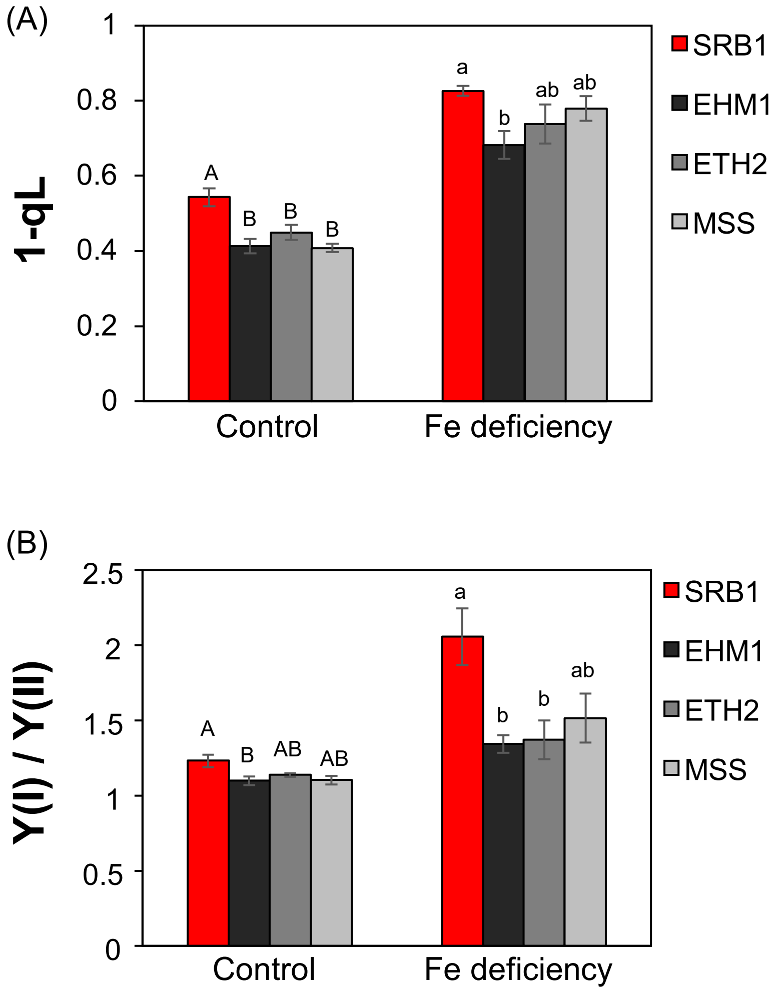

Fig. S2. The property of SRB1 associated with the reduction of the plastoquinone pool to a relatively high degree, a mechanism that allows photosystem I to avoid an over-reduced state (A) Redox state of plastoquinone pool (1-qL). SRB1 has the property of reducing the plastoquinone pool to a relatively high degree compared to other cultivars. (B) Ratio of the quantum yield of photosystem I relative to photosystem II [Y(I)/Y(II)], which indicate the electron flux in photosystem II and I. Data are represented as the means ± SE of three to four independent measurements. Different letters on individual columns when $p<0.05$ among varieties by Tukey's multiple test.

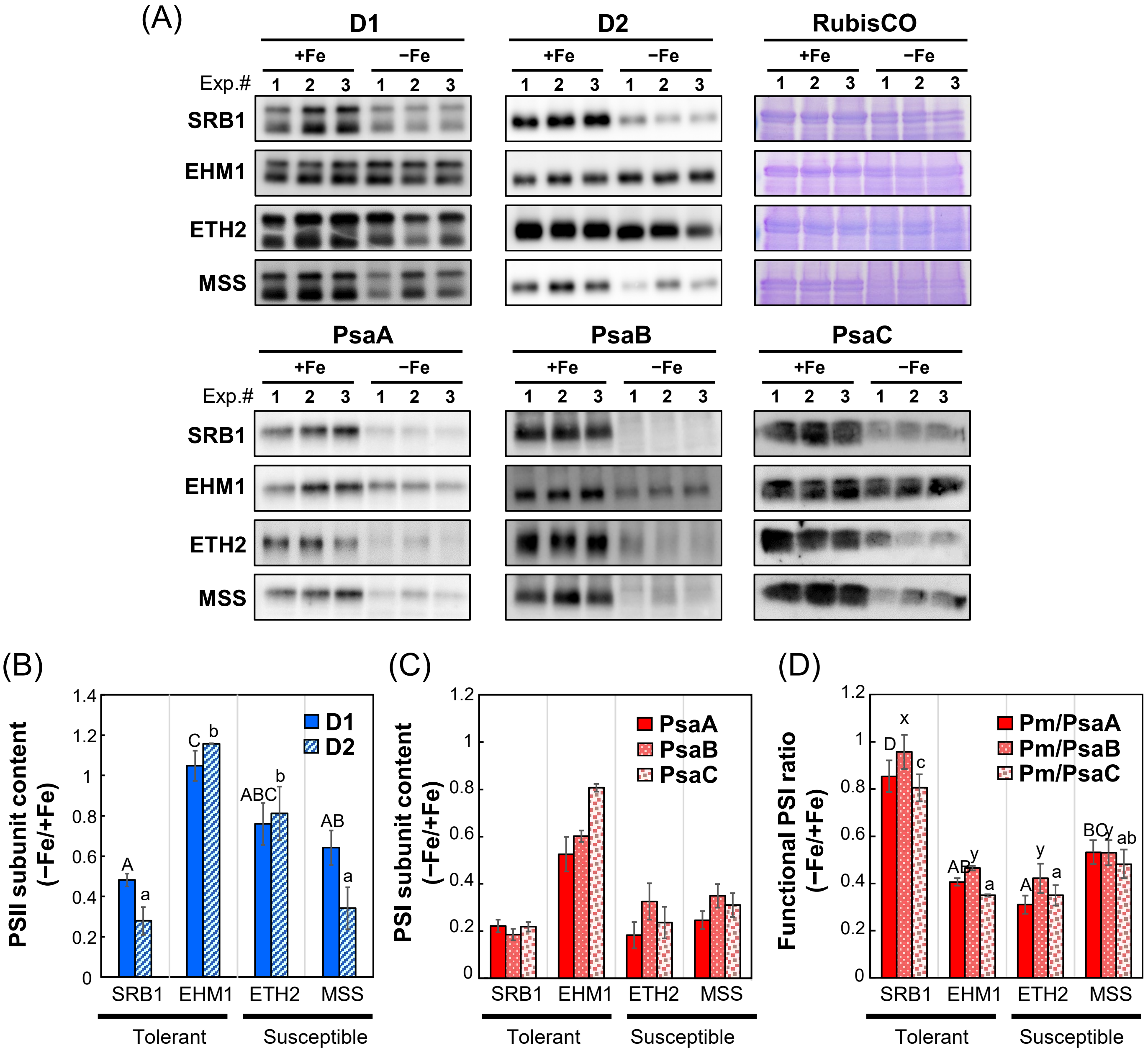

Fig.S3. Western blot analysis normalized on a per chlorophyll basis to compare the chlorophyll-binding thylakoid proteins in four barley cultivars with different Fe deficiency tolerance. (A) Immunoblot analysis of PSII reaction center proteins (D1, D2) and PSI reaction center proteins (PsaA, PsaB, and PsaC). CBB-stained RubisCO large subunits are shown as a loading control to show that the amount of protein in each lane of the triplicate is equal. Whole proteins extracted from leaves were separated by SDS-PAGE (each lane was loaded with 50 ng of chlorophyll for western blot analysis and 500ng of chlorophyll for CBB staining). Of these data, SRB1 and EHM1, and ETH2 match the data shown in Figure 5 in our previous report [7] and are identical to the sample lots used in the current paper.(B-C) The chemiluminescence of immunoblots detected with specific antibodies against each PSII subunit (D1 and D2) (B) and PSI subunit (PsaA, PsaB, and PsaC) (C) was quantified by Image J software and calculated as relative values for the Fe-sufficient condition when the Fe-sufficient condition was set as 1. (D) The retention rate of functional PSI under the Fe-deficient condition is expressed as the relative value of P700 maximum oxidation capacity (Pm) per PSI subunit content (value of Fig. S3C) to the Fe-sufficiency. Data are presented as means means ± SE of the data obtained from three independent leaves, with different letters on individual columns when $p<0.05$ among four barley varieties by Tukey multiple test.

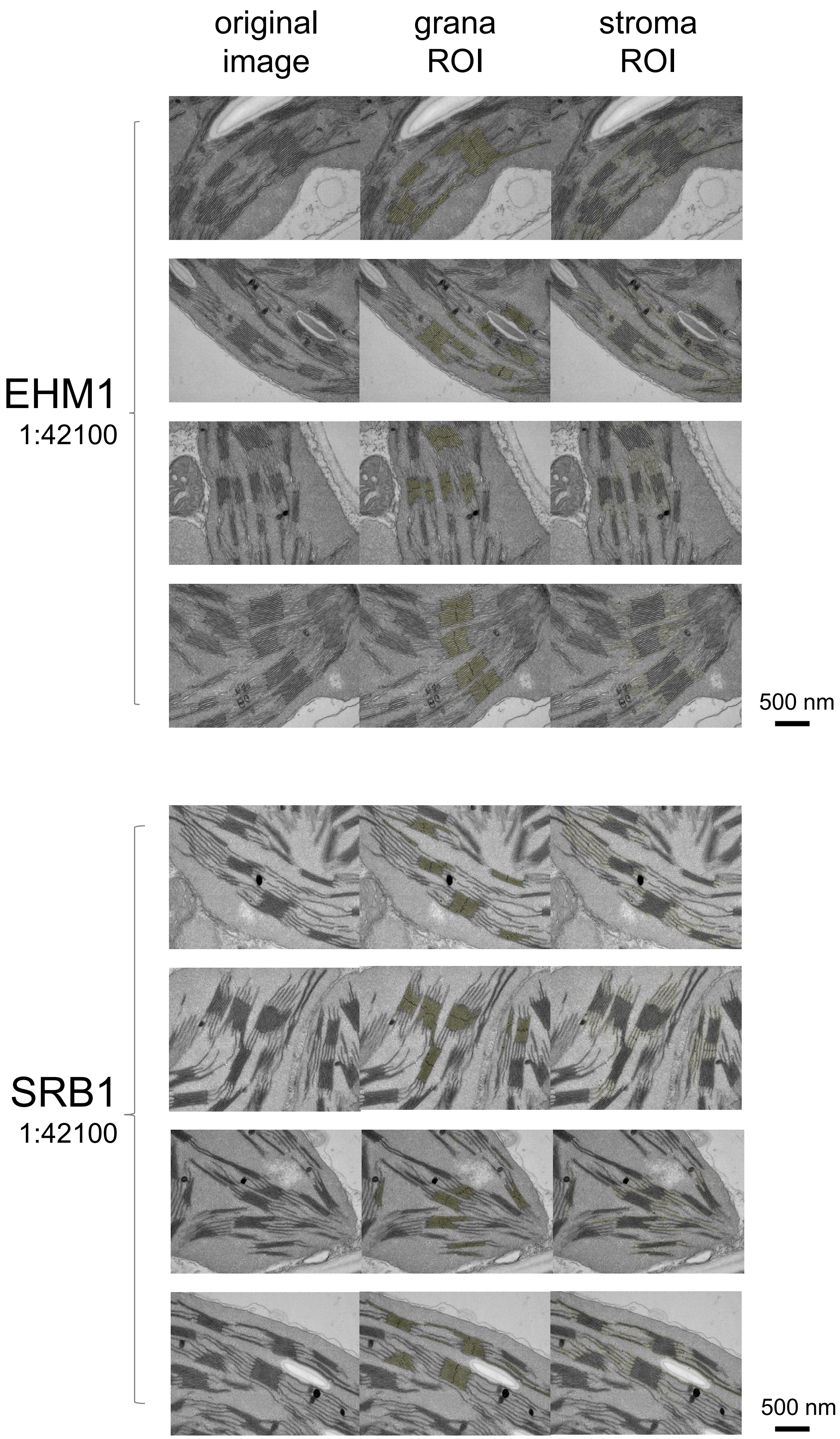

Fig.S4. Original TEM images and yellow tracing lines (regions of interest, ROIs) associated with Fig. 3.

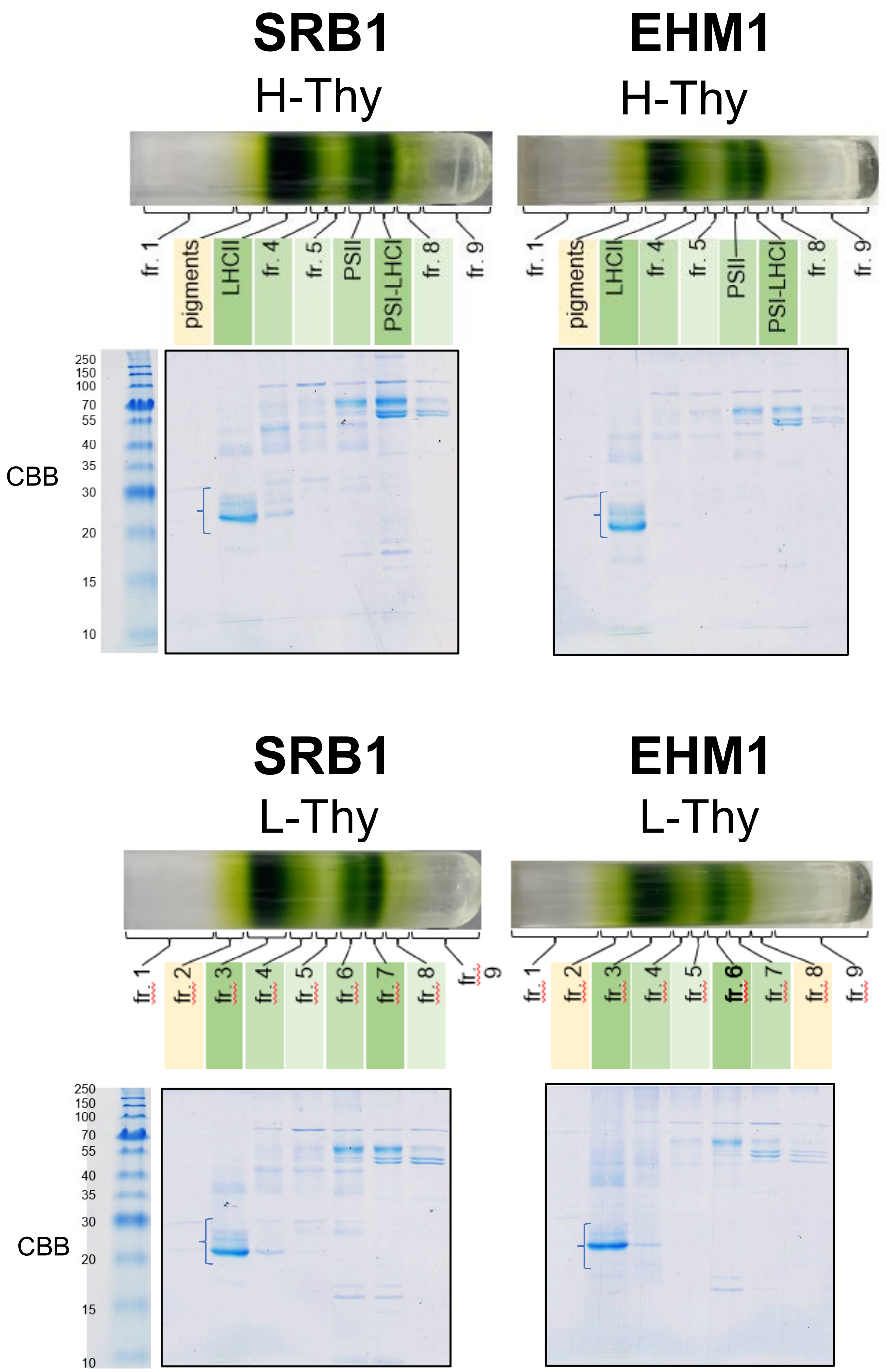

Fig.S5. CBB stain of gels applied with the same fractions in Fig. 4. Each lane was applied with 1/200 of each fraction. A brace indicates proteins of LHCII.

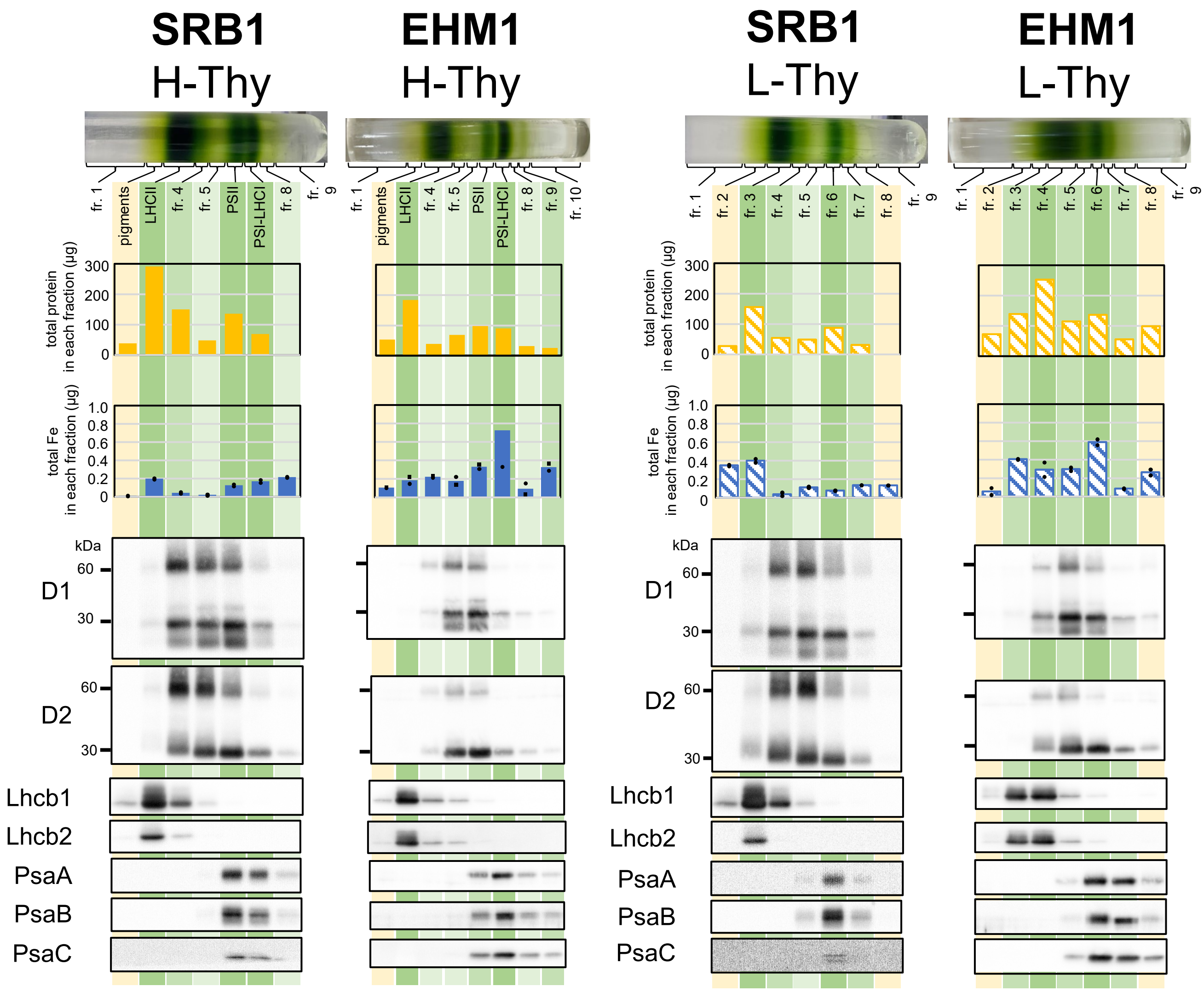

Fig.S6 Analyses of fractions obtained from SDG using the thylakoid membranes derived from frozen leaves. Details of analyses are the same as shown in Fig. 4. Fr. 4 and 5 of EHM H-Thy are corresponding to fr. 4 of SRB H-Thy.

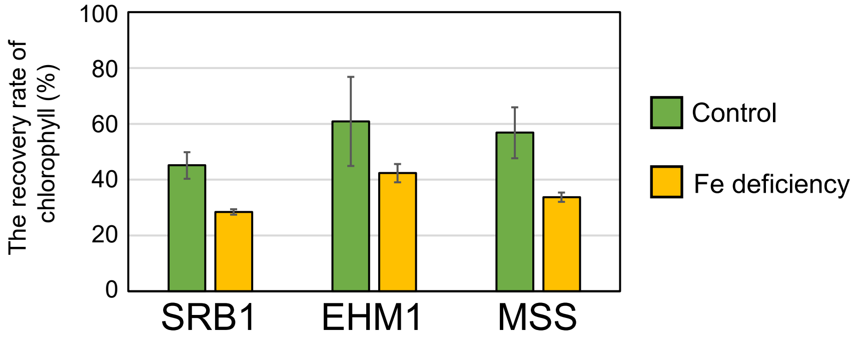

Fig.S7A. Supporting data for Figure 5. The recovery rate of chlorophyll in H-Thy and L-Thy from leaf chlorophyll. The mean values and S.E. of three experiments were shown.

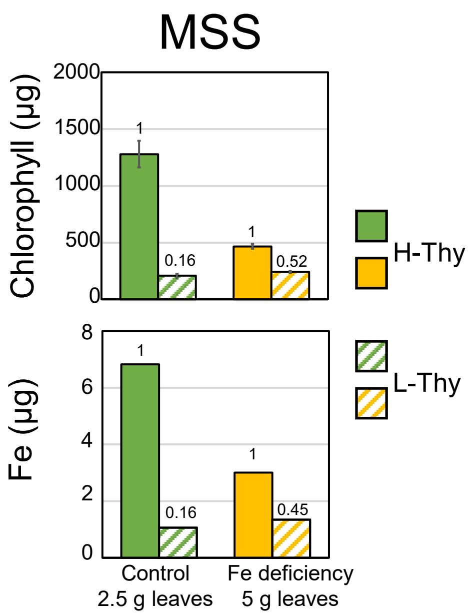

Fig.S7B. Supporting data for Figure 5. Chlorophyll (n=3) and Fe (n=1) contents of MSS corresponding to Figure 5. Fe-deficiency susceptible MSS was cultivated with Fe-deficient nutrient solution containing 3µM Fe to obtain enough amount of chlorotic leaves.

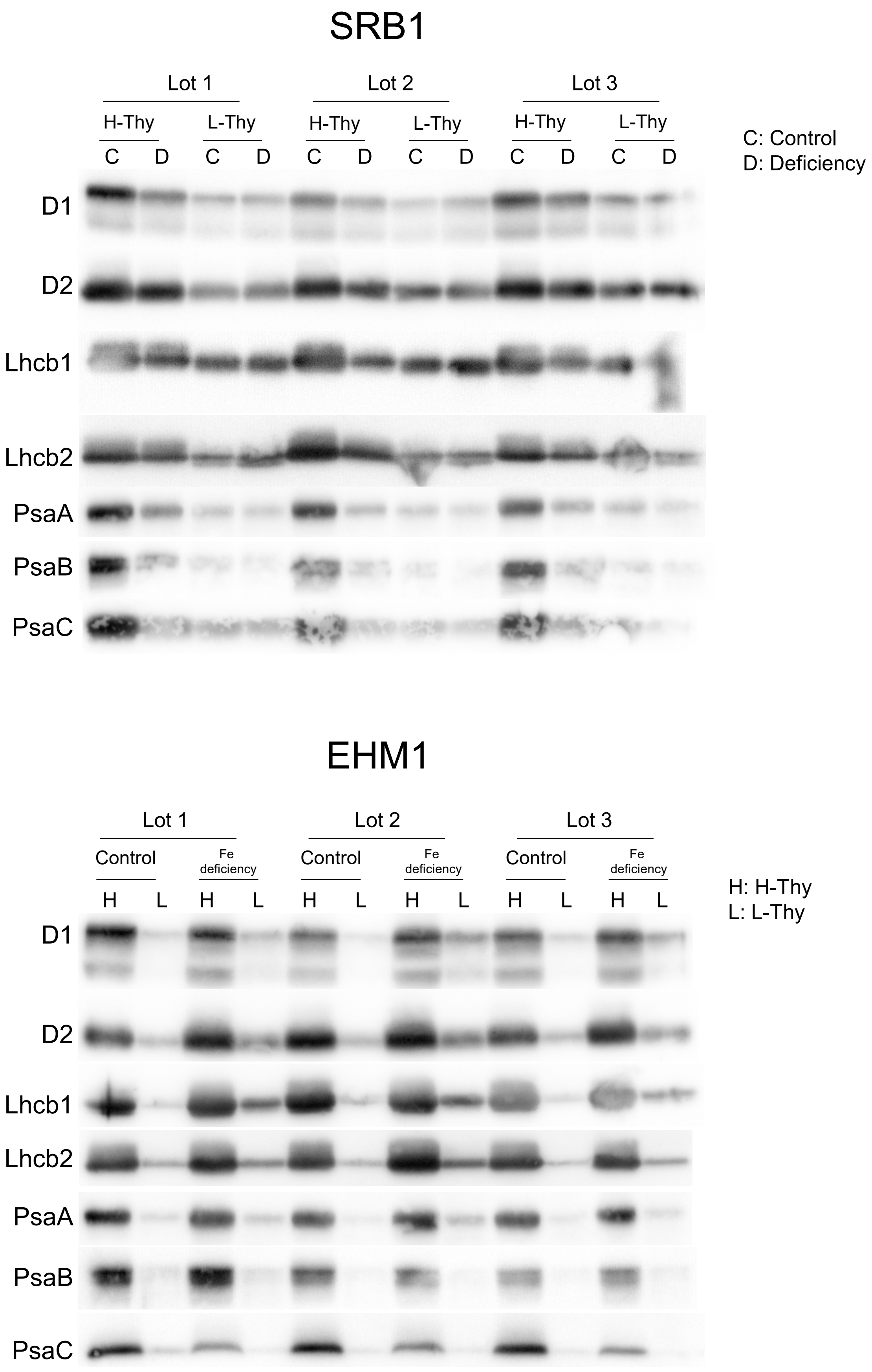

Fig.S8A. Original images of Western blot analysis shown in Fig. 5E.

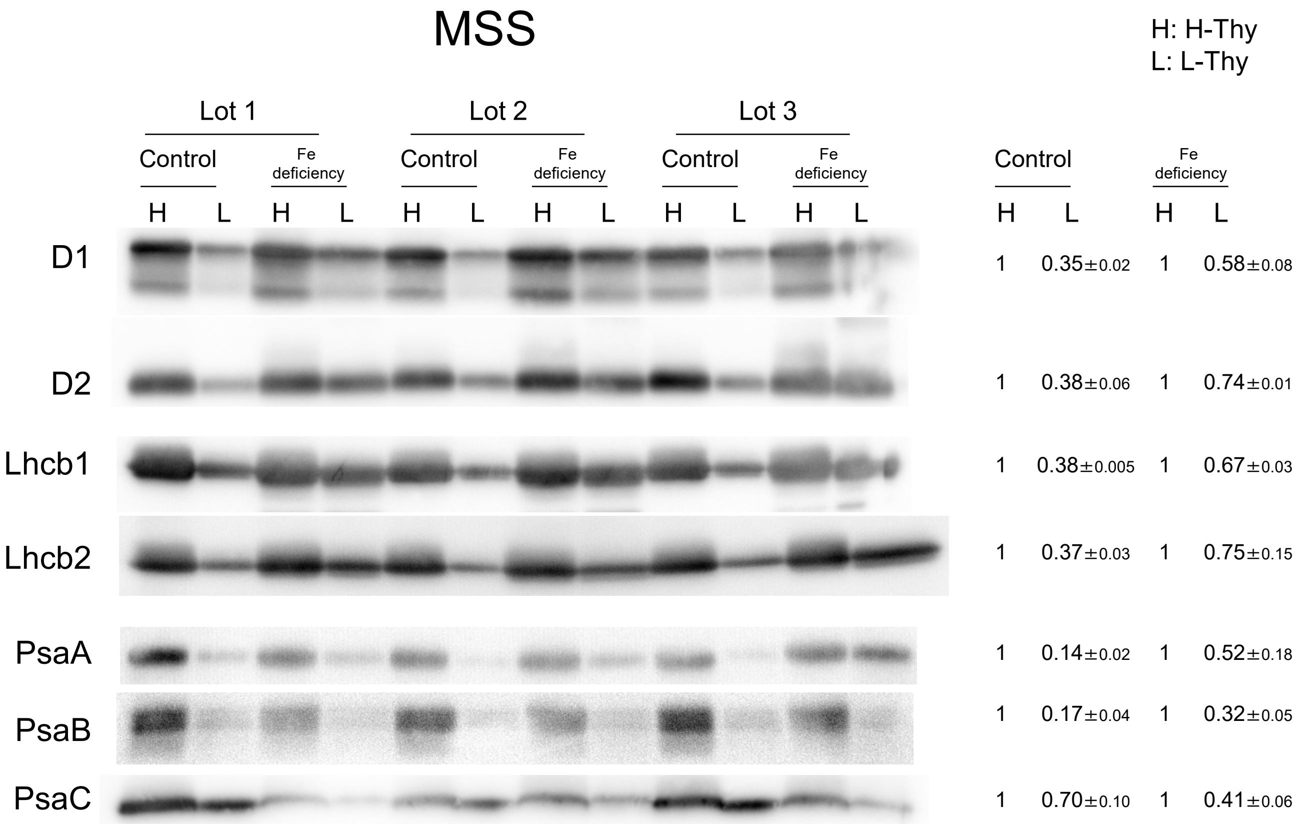

Fig.S8B. Western blot analysis derived from MSS corresponding to Fig. 5E.

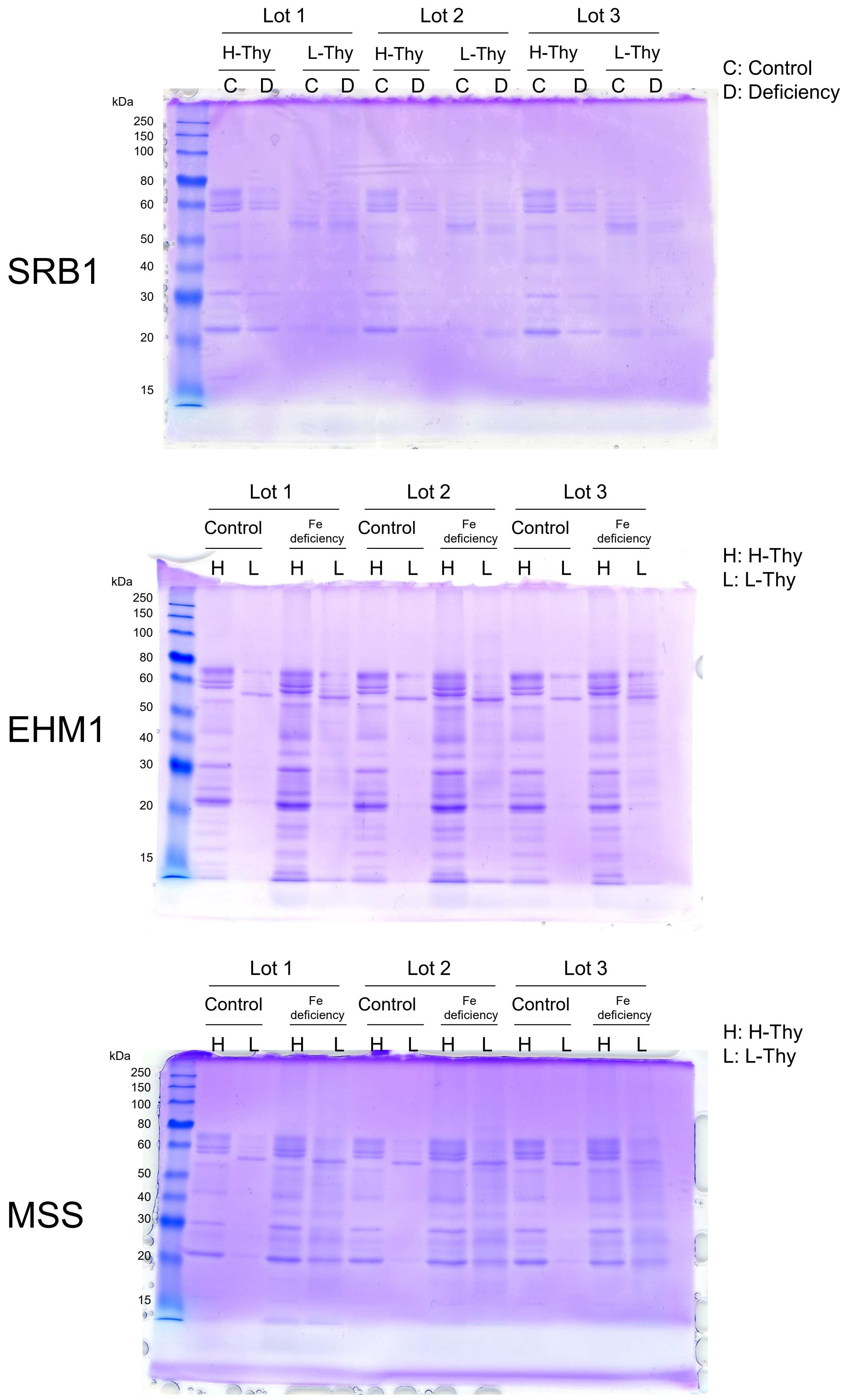

Fig.S8C. CBB stain images of corresponding Western blot analysis shown in Fig. 5E and Fig. S8B.

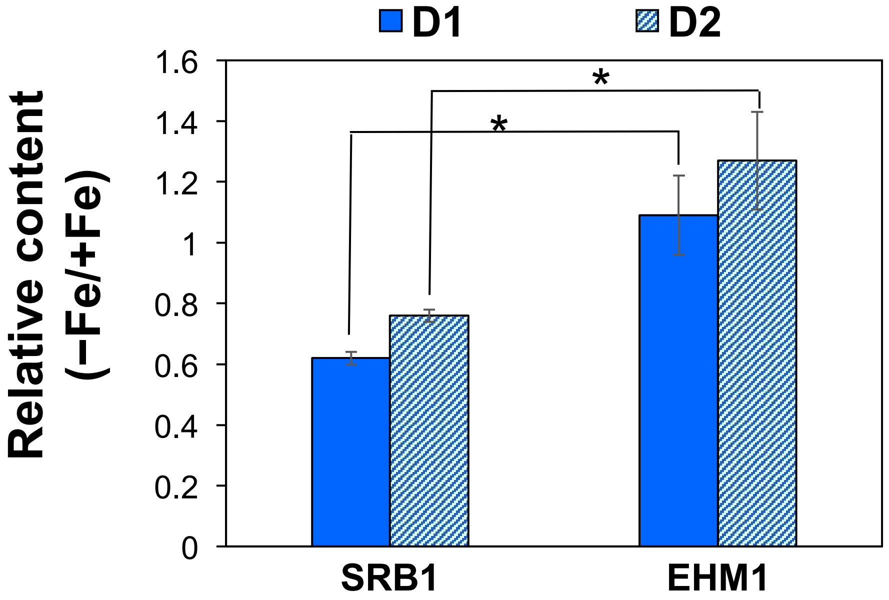

Fig.S9. Comparison of PSII core protein content among cultivars. Contents of D1 and D2 from data of H-Thy shown in Fig. 5 (E). Loading amount of Fe-deficient sample in Fig. 5 (E) was finally 8 folds of control sample as described in Fig. 5. Data are presented as means ± SE of the three independent experiments. Significant differences are shown by Student's *t*-test (* $p<0.05$).
